# Importance of Nodal Metastases Location in Pancreatoduodenectomy for Pancreatic Ductal Adenocarcinoma: Results from a Prospective, Lymphadenectomy Protocol

**DOI:** 10.1245/s10434-022-11417-3

**Published:** 2022-02-21

**Authors:** Giuseppe Malleo, Laura Maggino, Fabio Casciani, Gabriella Lionetto, Sara Nobile, Gianni Lazzarin, Salvatore Paiella, Alessandro Esposito, Paola Capelli, Claudio Luchini, Aldo Scarpa, Claudio Bassi, Roberto Salvia

**Affiliations:** 1 Department of Surgery, Dentistry, Gynecology and Pediatrics, Unit of General and Pancreatic Surgery, University of Verona, Verona, Italy; 2 Section of Pathology, Department of Pathology and Diagnostics, University of Verona, Verona, Italy

## Abstract

**Background:**

Implementing a prospective lymphadenectomy protocol, we investigated the nodal yields and metastases per anatomical stations and nodal echelon following upfront pancreatoduodenectomy (PD) for cancer. Next, the relationship between the extension of nodal dissection, the number of examined and positive nodes (ELN/PLN), disease staging and prognosis was assessed.

**Methods:**

Lymphadenectomy included stations 5, 6, 8a-p, 12a-b-p, 13, 14a-b, 17, and jejunal mesentery nodes. Data were stratified by N-status, anatomical stations, and nodal echelons. First echelon was defined as stations embedded in the main specimen and second echelon as stations sampled as separate specimens. Recurrence and survival analyses were performed by using standard statistics.

**Results:**

Overall, 424 patients were enrolled from June 2013 through December 2018. The median number of ELN and PLN was 42 (interquartile range [IQR] 34-50) and 4 (IQR 2-8). Node-positive patients were 88.2%. The commonest metastatic sites were stations 13 (77.8%) and 14 (57.5%). The median number of ELN and PLN in the first echelon was 28 (IQR 23-34) and 4 (IQR 1-7). While first-echelon dissection provided enough ELN for optimal nodal staging, the aggregate rate of second-echelon metastases approached 30%. Nodal-related factors associated with recurrence and survival were N-status, multiple metastatic stations, metastases to station 14, and jejunal mesentery nodes.

**Conclusions:**

First-echelon dissection provides adequate number of ELN for optimal staging. Nodal metastases occur mostly at stations 13/14, although second-echelon involvement is frequent. Only station 14 and jejunal mesentery nodes involvement was prognostically relevant. This latter station should be included in the standard nodal map and analyzed pathologically.

**Supplementary Information:**

The online version contains supplementary material available at 10.1245/s10434-022-11417-3.

Lymph node (LN) status is a well-established prognostic factor following pancreatoduodenectomy (PD) for pancreatic ductal adenocarcinoma (PDAC).^1^ The burden of nodal involvement is currently quantified based on the American Joint Committee on Cancer Staging (AJCC) TNM classification, whereby N1 status is defined as 1 to 3 positive LN (PLN) and N2 status as 4 or more PLN.^2–4^ In this framework, we have recently shown that pathologic examination of at least 28 regional LN ensures identification of 4 PLN with a 95% probability, avoiding underreporting of N2 patients.^5^ However, the issue of LN involvement might not be treated by the LN number solely, as the location of nodal metastases possibly impacts on tumor staging and patient prognosis.^6,7^ The extent of lymphadenectomy and the stations that should be removed in PD for PDAC were proposed in 2013 by the International Study Group of Pancreatic Surgery (ISGPS). The definition of “standard” dissection included stations 5, 6, 8a, 12b, 12c, 13, 14a-b, and 17 per the Japanese Pancreas Society nomenclature, although there was no general agreement on the exclusion of certain stations (i.e., 8p and 16b1).^8^ An internal discussion followed the ISGPS definition release, and a nodal dissection protocol was established at the unit of pancreatic surgery in Verona, entailing the ISGPS lymphadenectomy with extension to stations 8p, 12a-b, and jejunal mesentery nodes. This was not in contrast with the ISGPS principles, as dissection of stations situated near the “standard” basin and that could be easily incorporated into the resection plane was justified with general agreement.^8^ Following prospective application of this protocol, we herein examined the nodal yields and metastases per anatomical stations and the degree to which the extension of nodal dissection impacts on the number of ELN, PLN, and nodal staging. Finally, the prognostic role of these nodal parameters was investigated.

## Materials and Methods

### Lymph Node Dissection Protocol and Operative Details

**Fig. 1:**
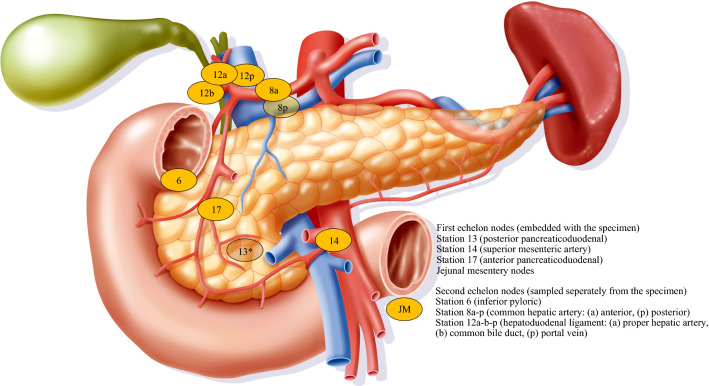
Institutional nodal dissection (anatomical stations and echelons) during PD for PDAC

The institutional lymphadenectomy protocol for was applied to upfront PD for presumed PDAC from June 2013. Dissection included stations 5, 6, 8a-p, 12a-b-p, 13, 14a-b, 17, and jejunal mesentery nodes per the Japanese Pancreas Society definition (Fig. 1).^9^ Station 16a2-b1 dissection was not routinely practiced, being these nodes sampled for frozen section at the surgeon’s discretion when macroscopically enlarged and/or suspicious. Because paraaortic node involvement was considered as distant metastatic disease, the resection was aborted in the instance of positive frozen section. For the purposes of this study, negative station 16a2-b1 samples were not included in the nodal count. The resection phase was performed as previously described.^10^ Superior mesenteric artery (SMA) dissection was generally conducted along its right aspect. Synchronous superior mesenteric vein-portal vein resection was performed in case of macroscopic vascular involvement. Arterial resection was not practiced in the upfront setting.

### Pathologic Examination

All specimens were analyzed by specialized pathologists. The slicing strategy consisted of a bivalve slicing followed by perpendicular slicing (4 mm). Stations 13 and 17 were examined following orange-peeling and full inclusion in paraffin blocks of peripancreatic soft tissues. Station 14 nodes were examined along the SMA groove. The first jejunal loop mesentery was fully included, and LN were identified microscopically. Other stations removed as distinct specimens were fully included and analyzed. The presence of LNs sliced in halves was always specified to avoid double counting. R-status was defined based on the presence of tumor cells within 1 mm from any resection margin. Tumors were staged according to the 8th edition of the AJCC manual from its introduction.^3^ For prior resections, tumors were restaged from the 7th to the 8th edition retrospectively.

### Exclusion Criteria

Only patients with conventional PDAC on final pathologic report were considered for the analysis. Other histotypes including cancers arising in the background of an intraductal papillary mucinous neoplasms, acinar cell carcinomas, squamous carcinomas, and rare variants were excluded. Patients receiving multivisceral resection, a macroscopically incomplete resection, and those who died within 90 days from the index operation also were excluded.

### Statistical Analysis

A precision-based approach was used to calculate sample size. Assuming that up to 35% of the subjects would harbor nodal metastases in stations removed as distinct specimens (based on the aggregate frequency of metastases at stations 6, 8, and 12 in a previous historical series^11^), and expecting a per protocol complete nodal dissection in 85% of cases, the study would require a sample size of 412 patients for estimating the expected proportion with 5% absolute precision and 95% confidence. Demographic, clinical, surgical, and pathologic details were prospectively collected in an electronic database. Continuous variables were expressed as medians with interquartile range (IQR). Categorical variables were presented as frequencies with percentages and compared using Chi-square test or Fisher’s exact test, as appropriate. Stations embedded in the PD specimen (13, 14a-b, 17, and jejunal mesentery LN) were defined as first nodal echelon, whereas stations sampled as distinct specimens (5, 6, 8a-p, 12a-b-p-c) were defined as second-echelon nodes. Total nodal yields and PLN per nodal echelon were then analyzed to establish their relative contribution to the staging process. The overall survival (OS) was calculated from the date of PD to the date of last follow-up or death. The recurrence-free survival (RFS) was calculated from the date of PD to the date of first recurrence, defined as the presence of biopsy-proven tumor or assumed based on serum Ca 19.9 levels and cross-sectional imaging studies, in conjunction with clinical picture. Cumulative event rates were calculated using the method of Kaplan and Meier, pairwise differences between survival and recurrence functions were evaluated using the log-rank test. Multivariable Cox regression models adjusted for relevant clinical and pathologic variables were constructed to explore the prognostic significance of node-related parameters, including the PLN-based system (N1/N2 per the current AJCC 8th edition definition),^3^ the number of metastatic stations, nodal echelons, and a LN station-based system. Each model concordance was evaluated using the Harrell’s c-statistics. The proportional hazard assumptions were verified. The *p*-values are presented with hazard ratios (HRs) and 95% confidence intervals (CI), as appropriate. Statistical significance was determined by a *p* value <0.05. Data were analyzed using SPSS software release 25 (SPSS, and IBM company, Armonk, NY), and R-software, version 3.6.3, (Foundation for Statistical Computing, Vienna, Austria, http://www.r-project.org).

## Results

### Analysis of Nodal Yields per Anatomical Stations and Nodal Echelons

In the study period (January 2013 to December 2018), 674 patients underwent PD for pathologically proven conventional PDAC. Of these, 231 patients were not eligible (228 received neoadjuvant therapy and 3 underwent PD with contiguous organ resection). Of the 443 eligible patients, 19 were excluded (7 received R2 resection, 2 had an oligometastatic disease, 10 died within 90 days from surgery). Therefore, the study population consisted of 424 patients (study flowchart in supplementary Fig. 1); their characteristics are displayed in supplementary Table 1. The median number of ELN was 42 (interquartile range [IQR] 34-50), the median number of PLN was 4 (IQR 2–8). Overall, 374 patients had nodal metastases (88.2%). Patient characteristics stratified by N-status and nodal echelons are displayed in Table 1. Overall, 248 patients (58.5%) harbored disease only in the first nodal echelon (stations 13, 14, 17, and jejunal mesentery nodes), 4 patients (0.9%) only in the second echelon (stations 5, 6, 8, 12), and 122 patients (28.8%) in both echelons. Therefore, the overall number of patients with metastases in the first and second nodal echelon was 370 (87.3%) and 126 (29.7%) respectively. The median number of ELN and PLN in the first echelon was 28 (IQR 23–34) and 4 (IQR 1–7). The addition of second-echelon nodes increased the median nodal count by 10 ELN (IQR 6–14) and 0 PLN (IQR 0-1), translating in only minor changes to the nodal staging (supplementary Table 2). In particular, 4 patients (0.9%) migrated from N0 to N1 status, 1 patient (0.02%) from N0 to N2 status, and 13 patients (3.0%) from N1 to N2 status. The rate of margin-positive resections progressively escalated with increasing nodal status and echelon involvement (both *p* < 0.001). Nonetheless, 43.1% of N2 patients and 38.1% of those with second echelon involvement received a R0 resection.

**Table 1:** Demographic, clinical, surgical, and pathologic details of the study population stratified by N-status and nodal echelons

	AJCC 8th N-status				Site of nodal metastases		
Parameter N (%)	N0 50 (11.8)	N1 135 (31.8)	N2 239 (56.4)	*p* value	First Echelon 248 (58.5)	Second Echelon 126 (29.7)	*p* value
*Sex*							
Female	21 (42.0)	63 (46.7)	112 (46.9)	0.815^*^	134 (54.0)	65 (51.6)	0.738^*^
Male	29 (58.0)	72 (53.3)	127 (53.1)	1.000^+^	114 (46.0)	61 (48.4)	0.735^°^
*Age (yr)*							
≤65	22 (44.0)	63 (46.7)	118 (49.4)	0.743^*^	122 (49.2)	59 (46.8)	0.767^*^
>65	28 (56.0)	72 (53.3)	121 (50.6)	0.693^+^	126 (50.8)	67 (53.2)	0.746^°^
*BMI*							
<25	31 (62.0)	82 (60.7)	147 (61.5)		155 (62.5)	74 (58.7)	
25–29	15 (30.0)	39 (28.9)	76 (31.8)		75 (30.2)	40 (31.7)	
>29	4 (8.0)	10 (7.4)	12 (5.0)	0.782^*^	12 (4.8)	10 (7.9)	0.774^*^
Missing	0 (0)	4 (3.0)	4 (1.7)	0.626^+^	6 (2.4)	2 (1.6)	0.596^°^
*ASA score*							
1	0 (0)	8 (5.9)	14 (5.9)		13 (5.2)	9 (7.1)	
2	41 (82.0)	97 (71.9)	182 (76.2)	0.364^*^	193 (77.8)	86 (68.3)	0.114^*^
3	9 (18.0)	30 (22.2)	43 (18.0)	0.605^+^	42 (16.9)	31 (24.6)	0.132^°^
*Preoperative pain*							
No	42 (84.0)	113 (83.7)	200 (83.7)	0.998^*^	203 (81.9)	110 (87.3)	0.402^*^
Yes	8 (16.0)	22 (16.3)	39 (16.3)	1.000	45 (18.1)	16 (12.7)	0.230^°^
*Preoperative jaundice*							
No	22 (44.0)	38 (28.1)	45 (18.8)	<0.001^*^	63 (25.4)	20 (15.9)	<0.001^*^
Yes	28 (56.0)	97 (71.9)	194 (81.2)	0.051^+^	185 (75.6)	106 (84.1)	0.049^°^
*Unintentional weight loss*							
No	25 (50.0)	59 (43.7)	103 (43.1)	0.666^*^	115 (46.4)	47 (37.3)	0.166^*^
Yes	25 (50.0)	76 (53.6)	136 (56.9)	0.996^+^	133 (53.6)	79 (62.7)	0.118^°^
*Diabetes mellitus*							
No	40 (80.0)	104 (77.0)	184 (77.0)	0.893^*^	191 (77.0)	97 (77.0)	0.893^*^
Yes	10 (20.0)	31 (23.0)	55 (23.0)	1.000^+^	57 (23.0)	29 (23.0)	1.000^°^
*Postoperative complications*							
No	21 (42.0)	60 (44.4)	108 (45.2)	0.918^*^	106 (42.7)	62 (49.2)	0.457^*^
Yes	29 (58.0)	75 (55.6)	131 (54.8)	0.976^+^	142 (57.3)	64 (50.8)	0.281^°^
*R–status*							
R0	42 (84.0)	88 (65.2)	103 (43.1)	<0.001^*^	143 (57.7)	48 (38.1)	<0.001^*^
R1	8 (16.0)	37 (34.8)	136 (56.9)	<0.001^+^	105 (42.3)	78 (61.9)	0.001^°^
*Tumor grade*							
G1	3 (6.0)	7 (5.2)	6 (2.5)		9 (3.6)	4 (3.2)	
G2	33 (66.0)	89 (65.9)	148 (61.9)		167 (67.3)	70 (55.6)	
G3	8 (16.0)	34 (25.2)	71 (29.7)	0.125^*^	59 (23.8)	46 (36.5)	0.032^*^
Others/missing	6 (12.0)	5 (3.7)	14 (5.9)	0.333^+^	13 (5.2)	6 (4.8)	0.081^°^
*Perineural invasion*							
No	2 (4.0)	2 (1.5)	1 (0.4)	0.095^*^	3 (1.2)	0 (0)	0.085^*^
Yes	48 (96.0)	133 (98.5)	238 (99.6)	0.296^+^	245 (98.8)	126 (100)	0.554^°^
*Lymphvascular Invasion*							
No	4 (8.0)	2 (1.5)	0 (0)	<0.001^*^	2 (0.8)	0 (0)	<0.001^*^
Yes	46 (92.0)	133 (98.5)	239 (100)	0.130^+^	246 (99.2)	126 (100)	0.552^°^
*Peripancreatic fat invasion*							
No	10 (20.0)	8 (5.9)	3 (1.3)	<0.001^*^	9 (3.6)	2 (1.6)	<0.001^*^
Yes	40 (80.0)	127 (94.1)	236 (98.7)	0.020^+^	239 (96.4)	124 (98.4)	0.347^°^
*T–status*							
T1	21 (42.0)	49 (36.3)	46 (19.2)		68 (27.4)	27 (21.4)	
T2	25 (50.0)	76 (56.3)	161 (67.4)		151 (60.9)	86 (68.3)	
T3	4 (8.0)	8 (5.9)	23 (9.6)	0.002^*^	22 (8.9)	9 (7.1)	0.154^*^
Missing	0 (0)	2 (1.5)	9 (3.8)	0.002^+^	7 (2.8)	4 (3.2)	0.526^°^
*N–status*							
N0	NA	NA	NA	NA	–	–	<0.001^°^
N1					118 (47.6)	17 (13.5)	
N2					130 (52.4)	109 (86.5)	
*No. positive LN stations*							
0	50 (0)	–	–	<0.001^+^	–	–	<0.001^°^
1	–	89 (65.9)	19 (7.9)		105 (42.3)	3 (2.4)	
2	–	41 (30.4)	87 (36.4)		108 (43.5)	20 (15.9)	
3	–	5 (3.7)	62 (25.9)		27 (10.9)	40 (31.7)	
≥4	–	0 (0)	71 (29.7)		8 (3.2)	63 (50.0)	
*Nodal metastases*							
N0	50 (0)	–	–	<0.001^+^	NA	NA	NA
First echelon	–	118 (87.4)	130 (54.4)				
Second echelon	–	17 (12.6)	109 (45.6)				
*Adjuvant treatment*							
No	6 (12.0)	18 (13.3)	28 (11.7)		27 (10.9)	19 (15.1)	
Yes	44 (88.0)	109 (80.7)	179 (74.9)	0.020^*^	193 (77.8)	95 (75.4)	0.109^*^
Missing	0 (0)	8 (5.9)	32 (13.4)	0.080^+^	28 (11.3)	12 (9.5)	0.427^°^

*p value for overall comparisons

^+^*p* value for N1 vs. N2

^°^*p* value for first versus second echelon

The nodal yields per anatomical station, the rate of metastatic involvement, the correlation with nodal staging (N1/N2) in node-positive patients, and the relative contribution of each nodal station to the number of ELN and PLN are reported in Table 2. The frequency of anatomical stations retrieval from fixed samples ranged from 76.2% (station 6) to 100% (station 14), excluding station 5 nodes, which were found in only 7% of fixed samples and were never metastatic. Hence, this station was excluded from further analyses. Stations with the highest median number of ELN were 13 (9 nodes) and 14 (7 nodes). These also were the most frequent metastatic sites (77.8% and 57.5% respectively).

**Table 2:** Nodal yields per anatomical lymph node stations in the overall cohort and correlation with nodal staging (N1/N2) in node-positive patients

	Examined LNs* median (IQR)	Positive LNs* median (IQR)	Frequency of harvesting and station involvement, n(%)	N1 n (%)	N2 n (%)	*p* value (N1 vs. N2)
*Station 5*	0.0 (0.0–0.0)	0.0 (0.0–0.0)	31/424 (7.3)			
5-			31/31 (100)	6 (100)	21 (100)	NC
5+			0/31 (0)	0 (0)	0 (0)	
*Station 6*	4.0 (1.0–6.0)	0.0 (0.0–0.0)	323/424 (76.2)			
6-			294/323 (91.0)	99 (98.0)	162 (85.7)	0.001
6+			29/323 (9.0)	2 (2.0)	27 (14.3)	
*Station 8#*	2.0 (1.0–5.0)	0.0 (0.0–0.0)	409/424 (96.5)			
8-			344/409 (84.1)	126 (96.9)	168 (73.4)	<0.001
8+			65/409 (15.9)	4 (3.1)	61 (26.6)	
*Station 12†*	2.0 (1.0–3.0)	0.0 (0.0–0.0)	380/424 (89.6)			
12-			302/380 (79.5)	107 (89.9)	148 (69.2)	<0.001
12+			78/380 (20.5)	12 (10.1)	66 (30.8)	
*Station 13*	9.0 (6.0–13.0)	2.0 (1.0–4.0)	423/424 (99.8)			
13-			94/423 (22.2)	33 (24.4)	12 (5.0)	<0.001
13+			329/423 (77.8)	102 (75.6)	227 (95.0)	
*Station 14*	7.0 (6.0–9.0)	1.0 (0.0–3.0)	424/424 (100)			
14-			180/424 (42.5)	84 (62.2)	46 (19.2)	<0.001
14+			244/424 (57.5)	51 (37.8)	193 (80.8)	
*Station 17*	6.0 (3.0–9.0)	0.0 (0.0–0.0)	399/424 (94.1)			
17-			318/399 (79.7)	114 (89.8)	157 (69.8)	<0.001
17+			81/399 (20.3)	13 (10.2)	68 (30.2)	
*Jejunal mesentery*	5.0 (2.0–8.0)	0.0 (0.0–0.0)	373/424 (88.0)			
Mesentery-			336/373 (90.1)	113 (96.6)	178 (84.4)	0.002
Mesentery+			37/373 (9.9)	4 (3.4)	33 (15.6)	

*LNs*, lymph nodes

*Evaluated in the overall cohort

^#^Includes stations 8a-p

*†*Includes stations 12a-b-p

### Survival and Recurrence Analysis

**Fig. 2:**
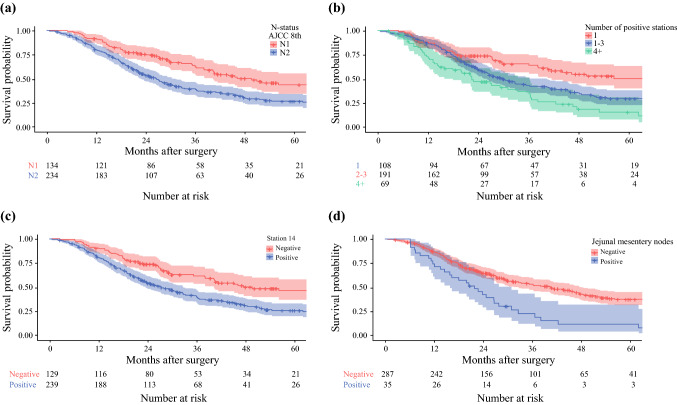
Kaplan-Meier curves of node-positive patients stratified by N-status (**A**), number of metastatic stations (**B**), station 14 (**C**) and jejunal mesentery node status (**D**)

Survival analysis was performed in 418 patients, after excluding 6 early-censored cases (within 6 months postoperatively). The median follow-up was 26.0 months (IQR 17.2–45.3) in the overall population and 39.4 months (95% confidence interval [CI] 23.9–59.6) in censored cases. The median OS was 36.0 months (95% CI 29.9–42.1). An overview of clinical-pathologic factors associated with survival in the whole study sample is presented in supplementary Table 3A. Univariable analysis of clinical-pathologic factors and nodal-related parameters associated with OS in node-positive patients is shown in supplementary Tables 4A and 3, respectively. Results of the multivariable analyses are summarized in Table 3: N2 status, a number of metastatic stations ≥4, metastases to station 14 and jejunal mesentery nodes, but not nodal echelons, were independently associated with survival in each model. The highest concordance was reached in the model including anatomic stations (c-index of 0.769). Survival curves stratified by these nodal-related parameters are shown in Fig. 2.

**Table 3:** Univariable analysis of the association between nodal-related parameters (N-status, lymph node station involvement, number of positive stations and nodal echelon) and survival or recurrence in node-positive patients

	Median overall survival months (95%CI)	*p* value	Median recurrence-free survival months (95%CI)	*p* value
*N-status*				
N1	49.73 (36.79–62.67)	**0.001**	31.000 (23.260–38.740)	**< 0.001**
N2	26.67 (22.77–30.56)		16.133 (12.982–19.284)	
*Station 5*				
5-	NA		NA	
5+				
*Station 6*				
6-	32.67 (24.58–40.75)	0.066	20.00 (16.73–23.27)	**0.013**
6+	27.133 (7.05–47.22)		13.07 (11.87–14.26)	
Station 8*				
8-	35.23 (28.24–42.22)	**0.017**	20.73 (16.79–24.67)	**0.003**
8+	31.03 (17.44–44.62)		13.80 (8.43–19.17)	
*Station 12†*				
12- 12+	35.37 (27.87–42.86) 31.47 (23.30–39.63)	0.746	20.27 (16.34–24.19) 19.00 (15.73–22.27)	0.140
*Station 13*				
13-	36.00 (17.95–54.05)	0.332	27.17 (14.50–39–83)	0.103
13+	32.73 (27.50–37.97)		18.97 (16.69–21.25)	
*Station 14*				
14-	49.73 (23.55–75.92)	**< 0.001**	27.43 (17.68–37.19)	**0.003**
14+	27.40 (22.53–32.27)		17.83 (15.11–20.56)	
*Station 17*				
17-	36.00 (27.87–44.12)	0.115	20.20 (16.22–24.18)	0.668
17+	26.47 (20.24–32.69)		19.00 (15.16–22.84)	
*Jejunal mesentery*				
Mesentery-	40.50 (32.31–48.69)	**< 0.001**	21.90 (18.60–25.20)	**< 0.001**
Mesentery+	21.90 (15.38–28.41)		12.00 (9.84–14.16)	
*Nodal metastases*				
First echelon	32.73 (25.21–40.26)	0.052	22.233 (16.846–27.621)	**0.010**
Second echelon	33.40 (24.86–41.94)		16.833 (12.096–21.571)	
*No. metastatic stations*				
1	73.27 (45.52–101.01)	**0.001**	30.43 (21.29–39.57)	**< 0.001**
2-3	30.0 (25.43–34.58)		19.10 (16.71–21.49)	
≥4	22.47 (15.63–29.30)		13.07 (11.54–14.59)	

Bold values indicate statistical significance

*Includes stations 8a-p

*†*Includes stations 12a-b-p

**Fig. 3:**
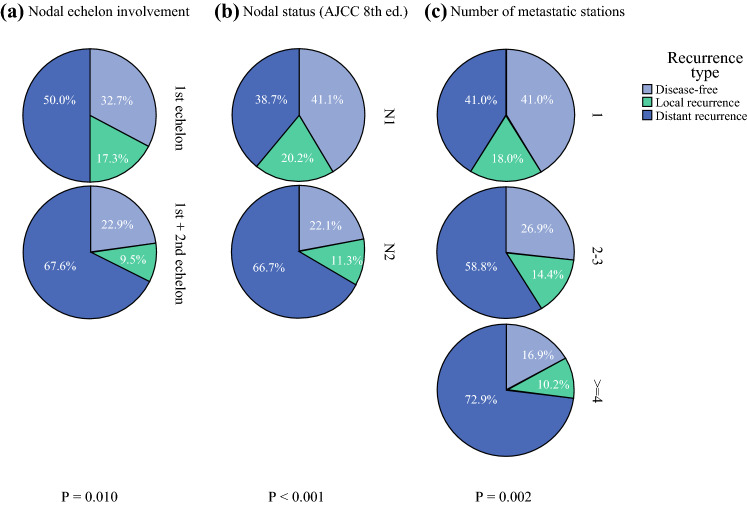
Recurrence status of node-positive patients stratified by nodal echelon involvement (**A**), nodal status (**B**), and number of metastatic stations (**C**)

Information on recurrence site was available in 368 patients (88.0%). Of the 249 patients who recurred (67.7%), 52 experienced an isolated local recurrence (14.1%), whereas 197 developed distant metastases (53.5%). The median RFS was 20.7 months (95% CI 17.7-23.7). Clinical-pathologic factors associated with recurrence in the whole sample are summarized in supplementary Table 3B. Median RFS of node-positive patients stratified by clinical-pathologic factors and nodal-related parameters is shown in supplementary Table 4B and Table 4, respectively. Notably, all nodal-related parameters were associated with RFS. The set of multivariable models was replicated for the independent association with disease recurrence: N2 status, a number of metastatic stations ≥4, metastases to station 14, 12 and jejunal mesentery nodes, but not nodal echelons, were independently associated with RFS in each model (Table 5). The highest concordance was again reached in the model including anatomical stations (c-index of 0.709). Analysis of first recurrence site showed and incremental increase in the rate of distant recurrence with increasing nodal involvement, either in terms of PLN, number of positive stations, and nodal echelons (Fig. 3).

**Table 4:** Multivariable analysis of factors associated with survival in the subset of node-positive patients

Variables	Model 1 N-status c-index: 0.699		Model 2 Lymph node stations c-index: 0.769		Model 3 Number of positive stations c-index: 0.705		Model 4 Nodal echelons c-index: 0.679	
	Hazard ratio (95% CI)	*p* value	Hazard ratio (95% CI)	*p* value	Hazard ratio (95% CI)	*p* value	Hazard ratio (95% CI)	*p* value
*Vascular resection*								
No	1		1		1		1	0.020
Yes	1.626 (1.082–2.445)	0.019	2.357 (1.291–4.305)	0.005	1.725 (1.149–2.589)	0.009	1.603 (1.076–2.389)	
*Tumor grade*								
G1	1		1		1		1	
G2	1.786 (0.641–4.975)	0.626	3.030 (0.507–30.623)	0.233	1.834 (0.663–5.077)	0.243	2.054 (0.750–5.622)	0.161
G3	4.396 (1.548–10.487)	0.005	10.182 (1.281–20.933)	0.008	4.059 (1.435–11.48)	0.008	4.979 (1.786–12.640)	0.002
*N-status*								
N1	1		NA	–	NA	–	NA	–
N2	1.958 (1.368–2.803)	<0.001						
*Station 14*								
14-	NA	–	1		NA	–	NA	–
14+			2.372 (1.384–4.066)	0.002				
*Jejunal mesentery nodes*								
jejunal-	NA	–	1		NA	–	NA	–
jejunal+			3.927 (2.053–7.509)	< 0.001				
*No. positive stations*								
1	NA	–	NA	–	1		NA	–
2–3					1.476 (0.900–2.422)	0.123		
≥4					2.449 (1.370–3.693)	0.001		
*Adjuvant treatment*								
No	1		1		1		1	
Yes	0.432 (0.279–0.669)	<0.001	0.306 (0.175–0.535)	<0.001	0.473 (0.306–0.730)	0.001	0.508 (0.331–0.779)	0.002

**Table 5:** Multivariable analysis of factors associated with recurrence-free survival in the subset of node-positive patients

Variables	Model 1 N-status c-index: 0.663		Model 2 Lymph node stations c-index: 0.709		Model 3 Number of positive stations c-index: 0.661		Model 4 Nodal echelons c-index: 0.632	
	Hazard ratio (95% CI)	*p* value	Hazard ratio (95% CI)	*p* value	Hazard ratio (95% CI)	*p* value	Hazard ratio (95% CI)	*p* value
*T-status*								
T1	1		1		1		1	
T2	1.680 (1.176–2.402)	0.004	1.660 (0.995–2.772)	0.052	1.763 (1.232–2.522)	0.002	1.664 (1.168–2.372)	0.005
T3	2.121 (1.168–3.853)	0.014	1.146 (0.316–4.148)	0.836	2.041 (1.116–3.733)	0.0210	2.261 (1.242–4.115)	0.008
*N-status*								
N1	1		NA	–	NA	–	NA	–
N2	1.572 (1.143–2.162)	0.005						
Station 12*								
12-	NA	–	1		NA	–	NA	–
12+			1.896 (1.302–1.921)	0.024				
*Station 14*								
14-	NA	–	1		NA	–	NA	–
s14+			1.774 (1.100–2.861)	0.019				
*Jejunal mesentery nodes*								
jejunal-	NA	–	1		NA	–	NA	–
jejunal+			3.740 (1.983–7.054)	<0.001				
*No. positive stations*								
1	NA	–	NA	–	1		NA	–
2–3					1.225 (0.862–1.742)	0.258		
≥4					1.816 (1.149–2.871)	0.011		
*Adjuvant treatment*								
No	1	0.047	1	0.001	1	0.072	1	0.129
Yes	0.627 (0.395–0.994)		0.370 (0.204–0.671)		0.656 (0.415–1.039)		0.703 (0.445–1.109)	

*Includes stations 12a-b-p

## Discussion

Employing an institutional lymphadenectomy protocol in upfront PD for PDAC, we investigated prospectively the nodal yields per anatomical stations and nodal echelons, and assessed the impact of metastases location on staging, disease recurrence, and survival. Specimen dissection along the right hemi-circumference of the SMA and complete soft tissue clearance along the hepatoduodenal ligament, common hepatic artery and pyloric area resulted in a median of 42 ELN (IQR 34-50), a substantially greater figure relative to other PD series.^1–4^ In turn, this translated to a median number of 4 PLN (IQR 2-8), a rate of node-positive patients of 88.2%, and a rate of N2 disease of 56.4%. There was a large variation in the number of ELN for each station and a significant number of cases where no LN were retrieved from certain anatomic locations (i.e., station 5). This is consistent with autopsy studies assessing the number of regional LN in sites relevant to lymphadenectomy in gastric cancer, showing striking individual differences either in the total number of LN and number of single stations.^12–14^ In the present series, nodal metastases most frequently occurred along the SMA groove (57.5%) and posteriorly to the pancreatic head (77.8%), two anatomic stations embedded with the main specimen and thus included in the first nodal echelon.^15^ As per other first-echelon stations, anterior pancreaticoduodenal nodes were metastatic in around 20% of the cases and—surprisingly—metastases to jejunal mesentery nodes were found in 10% of patients. Although jejunal mesentery nodes are not included in any PDAC nodal map and the “tail” of the duodenum is normally removed from the specimen before slicing, the jejunal mesentery is routinely included in paraffin blocks by our pathologists, because surgical dissection is conducted along jejunal pedicles and not tangentially to the jejunal loop. Indeed, jejunal lymph nodes are not searched for surgically but found microscopically at the time of pathologic examination.

Overall, the median number of ELN in the first nodal echelon was 28, equaling the optimal threshold for an adequate N2 staging suggested by a recent joint analysis from the authors’ institution and the Massachusetts General Hospital group.^5^ This once again demonstrates that high nodal yields are not necessarily the result of an extended lymphadenectomy, but rather the effect of a thorough pathologic examination with orange-peeling and full inclusion of peripancreatic tissues.^16,17^ The second nodal echelon increased the overall median nodal count by 10 ELN (IQR 6-13). Modeling a staging process with or without the second echelon did not lead to a tangible improvement in nodal classification, as only 18 patients (4.2%) would have been upstaged adding the second echelon. Nonetheless, the rates of metastases at pyloric nodes, nodes around the common hepatic artery and the hepatoduodenal ligament were 9%, 16%, and 21%, resulting in a 30% aggregate rate of second echelon involvement. This is in sharp contrast with previous studies that analyzed separately second-echelon LN, where approximately only 10% of patients with resectable PDAC harbored disease at this level.^15,18,19^ Indeed, the concept of second echelon nodes is ill defined in the surgical literature, depending on each institution’s nodal dissection policies. In most papers, the second echelon included LN within the retroperitoneum anterior to the inferior vena cava or aorta. This set of nodes was not part of our standard dissection protocol, with station 16a2-b1 being harvested for frozen section analysis in very selected cases, when macroscopically enlarged and/or suspicious. The finding of nodal involvement at this level was considered as distant metastasis, contraindicating the planned resection procedure.^20–22^ Instead, we dissected systematically stations 8a-p and 12a-b-p, because nodes along the common bile duct, hepatic artery, and portal vein are anatomically part of a lymphatic network that rings the pancreas, for which prespecified draining routes have not been identified by anatomical studies.^23–25^ Furthermore, no clear anatomical demarcation lines exist between regions that drain upwards to the hepatoduodenal ligament and downward to the SMA. Besides these anatomical concepts, removal of stations 8 and 12 allows for identification, encirclement, and control of the peripancreatic vasculature during the resection phase, thereby increasing surgical safety especially in the instance of complex vascular dissection. Indeed, extending the dissection beyond the standard boundaries to nearby stations that can be easily incorporated into the resection plane was justified by ISGPS members, with general agreement.^8^ Nonetheless, opponents of systematic nodal dissection in PDAC may still argue that our protocol involves a somewhat “extended” lymphadenectomy, something that has not been associated with improved survival in randomized controlled trials.^26,27^ The present study was not designed to investigate whether retrieval of second-echelon nodes improves survival, because all patients received an identical lymphadenectomy, established *a priori*. As remarked above, the frequency of second nodal echelon involvement was as high as 30%, with 38% of these patients receiving a R0 resection, representing 11.3% of the general population.

On multivariable models, N-status, an increasing number of metastatic stations, and metastases to station 14 and jejunal mesentery nodes were associated with survival. While the prognostic role of station 14 had been already shown in a previous retrospective analysis of nonconsecutive patients matching the ISGPS lymphadenectomy,^11^ the concept of jejunal mesentery nodal station is novel and of relevance given the 5-year survival rate of only 7.6% in the instance of its metastatic involvement. Recurrence analysis showed analogous results, adding station 12 to the variable set associated with disease relapse. Interestingly, there was an incremental increase in the rate of distant recurrence in N2 patients, in those with metastases to second-echelon nodes and in those with and increasing number of metastatic stations, confirming that worsening nodal parameters serve as a proxy of more aggressive disease.

On the ground of these data, we are far from claiming that our lymphadenectomy protocol has a measurable, causative effect on recurrence and survival, although we believe that a diligent nodal dissection serves as an indicator of surgical quality and plays an integral role in the PDAC treatment trajectory, together with multidisciplinary management, patient selection, and access to adjuvant therapy. Remarkably, the median survival rate of 36 months (5-year survival rate of 32.5%), and the median recurrence-free survival of 20 months herein reported compare favorably with other contemporary large series.^28–30^

This analysis has several major limitations. The sample size was calculated to estimate the aggregate proportion of nodal metastases in the second echelon, and not the frequency of metastases per single station, thereby requiring regrouping for certain analyses (i.e., stations 8 and 12). Another limitation is that the analysis was done only on patients undergoing upfront PD, and thus these results cannot be extrapolated for those undergoing PD following neoadjuvant treatment. This will be the focus of a separate work.

## Conclusions

Applying a prospective protocol of nodal dissection in upfront PD for PDAC, the overall number of ELN was 42, with a node-positive rate of 88.2% and a rate of N2 disease of 56.4%. Nodal metastases occurred more frequently within the surgical specimen, in stations 13 (77.8%) and 14 (57.5%). The median number of ELN in the first nodal echelon was 28, demonstrating that an adequate nodal count for optimal staging does not require extended dissection and can be achieved through a diligent pathological examination. Examining nodes in the second echelon does not improve the staging process substantially. Nonetheless, the aggregate frequency of metastases in stations removed as distinct specimens approached 30%, despite metastases in the second echelon did not result to be independently associated with recurrence and survival. Conversely, N-status per the AJCC 8th edition criteria, an increasing number of metastatic stations as well as metastases to station 14 and to jejunal mesentery nodes were prognostically relevant. The jejunal mesentery should be dissected tracing down the first-order jejunal pedicles; and jejunal nodes should be included in the standard nodal map and analyzed pathologically.

## Supplementary Information

Below is the link to the electronic supplementary material.

Supplementary file1 (DOCX 31 kb)
