# Supplementary material for: Importance of Nodal Metastases Location in Pancreatoduodenectomy for Pancreatic Ductal Adenocarcinoma: Results from a Prospective, Lymphadenectomy Protocol
Source: Ann Surg Oncol. 2022 Feb 21;29(6):3477–88. doi: 10.1245/s10434-022-11417-3 (PMC9072462; doi:10.1245/s10434-022-11417-3)
Supplement: Supplementary file 1 — Supplementary file1 (DOCX 31 kb) [file 10434_2022_11417_MOESM1_ESM.docx]

**Supplementary table 1.** Demographic, clinical, surgical, and pathologic details of the study population

| **Parameter** | **Total, n (%)**  424 (100%) |
| --- | --- |
| **Sex**  Female  Male | 196 (46.2)  228 (53.8) |
| **Age**  ≤ 65  > 65 | 203 (47.9)  221 (52.1) |
| **BMI**  < 25  25 – 29  > 29  Missing | 260 (61.3)  130 (30.7)  26 (6.1)  8 (1.9) |
| **ASA score**  1  2  3 | 22 (5.2)  320 (75.5)  82 (19.3) |
| **Preoperative pain**  No  Yes | 355 (83.7)  69 (16.3) |
| **Preoperative jaundice**  No  Yes | 105 (24.8)  319 (75.2) |
| **Unintentional weight loss**  No  Yes | 187 (44.1)  237 (55.9) |
| **Diabetes mellitus**  No  Yes | 328 (77.4)  96 (22.6) |
| **Postoperative complications**  No  Yes | 189 (44.6)  235 (55.4) |
| **R-status**  R0  R1 | 233 (55.0)  191 (45.0) |
| **Tumor grade**  G1  G2  G3  Others/Missing | 16 (3.8)  270 (63.7)  113 (26.7)  25 (5.9) |
| **Perineural invasion**  No  Yes | 5 (1.2)  419 (98.8) |
| **Lymphvascular Invasion**  No  Yes | 6 (1.4)  418 (98.6) |
| **Peripancreatic fat invasion**  No  Yes | 21 (5.0)  403 (95.0) |
| **T-status**  T1  T2  T3  Missing | 116 (27.4)  262 (61.8)  35 (8.3)  11 (2.6) |
| **N-status**  N0  N1  N2 | 50 (11.8)  135 (31.8)  239 (56.4) |
| **No. of positive LN stations**  0  1  2  3  ≥4 | 50 (11.8)  108 (25.5)  128 (30.2)  67 (15.8)  71 (16.7) |
| **Nodal metastases**  N0  First echelon  Second echelon | 50 (11.8)  248 (58.5)  126 (29.7) |
| **Adjuvant treatment**  No  Yes  Missing | 52 (12.3)  332 (78.3)  40 (9.4) |

**Supplementary table 2.** Distribution of nodal classes (AJCC 8th edition) modeling a staging process with and without the second nodal echelon.

|  | **Extension of nodal dissection (n=424)** | |
| --- | --- | --- |
| **N-status** | **First nodal echelon**  **(13,14,17, jejunal mesentery)** | **First and second nodal echelon**  **(13,14,17, jejunal mesentery, 6,8,12)** |
| N0 | 55 (12.9%) | 50 (11.8%) |
| N1 | 145 (34.1%) | 136 (32.1%) |
| N2 | 224 (53.0%) | 238 (56.1%) |

**Supplementary table 3**. Factors associated with survival (A) and recurrence (B) in the whole study sample.

| **Variable** | 1. **Survival Analysis** | | 1. **Recurrence Analysis** | |
| --- | --- | --- | --- | --- |
|  | **Median OS, months (95% CI)** | **p-value** | **Median DFS, months (95% CI)** | **p-value** |
| **Sex**  Male  Female | 32.767 (25.060-40.473)  36.700 (27.643-45.757) | 0.868 | 21.467 (17.596-25.337)  20.267 (15.794-24.739) | 0.364 |
| **Preoperative pain**  No  Yes | 35.367 (28.772-41.962)  38.233 (22.940-53.527) | 0.471 | 20.600 (17.607-23.593)  24.500 (10.188-38.812) | 0.151 |
| **Unintentional weight loss**  No  Yes | 32.767 (24.181-41.352)  36.367 (27.054-45.679) | 0.415 | 19.833 (17.241-22.425)  23.000 (18.163-27.837) | 0.641 |
| **Preoperative jaundice**  No  Yes | 40.700 (22.832-58.568)  34.433 (28.393-40.474) | 0.372 | 33.200 (22.882-43-518)  19.000 (17.045-20.955) | **0.002** |
| **Preoperative vascular involvement**  No  Yes | 40.300 (32.424-48.176)  27.400 (19.552-35.248) | **0.009** | 23.767 (19.513-28.020)  18.600 (14.103-23.097) | **0.032** |
| **Diabetes mellitus**  No  Yes | 35.367 (29.036-41.697)  31.933 (24.695-39.172) | 0.996 | 20.733 (17.321-24.146)  20.400 (14.093-26.707) | 0.822 |
| **ASA score**  1  2  3 | 63.867 (45.027-82.706)  32.733 (26.098-39.369)  35.233 (22.579-47.888) | **0.050** | 24.000 (13.274-34.726)  20.200 (16.557-23.843)  20.267 (13.623-26.910) | 0.978 |
| **R-status**  R0  R1 | 47.500 (38.491-55.509)  27.533 (22.422-36.645) | **<0.001** | 27.433 (20.428-34.439)  18.567 (16.128-21.005) | **0.001** |
| **Tumor grade**  G1  G2  G3 | NC  44.033 (33.389-54.677)  19.000 (16.043-21.957) | **<0.001** | 62.633 (14.251-111.016)  23.767 (19.314-28.219)  15.067 (10.215-19.918) | **0.001** |
| **Perineural invasion**  No  Yes | NC  35.367 (29.568-41.166) | 0.246 | 46.067 (N  20.400 (17.326 23.474) | 0.169 |
| **Lymphvascular Invasion**  No  Yes | NC  35.367 (29.567-41.166) | 0.510 | 46.067 (NC)  20.600 (17.477-23.723) | 0.583 |
| **Peripancreatic fat invasion**  No  Yes | 45.700 (33.865-57.535)  34.433 (28.506-40.361) | 0.422 | 34.300 (29.333-39.267)  20.100 (17.172-23.028) | 0.118 |
| **Postoperative complications**  No  Yes | 49.733 (35.711-63.756)  33.400 (27.409-39.391) | 0.086 | 23.000 (19.090-26.910)  20.000 (16.274-23.726) | 0.776 |
| **Adjuvant treatment**  No  Yes | 23.500 (10.048-36.952)  41.433 (34.406-48.461) | **0.001** | 17.500 (10.742-24.258)  21.900 (18.643-25.157) | 0.167 |
| **T-status**  T1  T2  T3 | 44.067 (33.010-55.123)  32.767 (26.684-38.849)  20.800 (13.737-27.863) | **0.004** | 33.133 (24.277-41.990)  19.567 (17.425-21.708)  14.700 (2.741-26.659) | **<0.001** |
| **N-status**  N0  N1  N2 | 51.900 (46.499-57.301)  49.733 (36.792-62.675)  26.667 (22.771-30.562) | **<0.001** | 35.367 (20.038-50.696)  31.000 (23.260-38.740)  16.133 (12.982-19.284) | **<0.001** |
| **Number of metastatic stations**  0  1  2-3  ≥4 | 51.900 (46.499-57.301)  73.267 (45.522-101.011)  30.0 (25.426-34.574)  22.467 (15.632-29.301) | **<0.001** | 35.367 (20.038-50.696)  30.433 (21.290-39.576)  19.100 (16.607-21.493)  13.067 (11.539-14.594) | **<0.001** |
| **Nodal metastases**  N0  First echelon  Second echelon | 51.900 (46.499-57.301)  32.733 (25.210-40.257)  33.400 (24.860-41.940) | **<0.001** | 35.367 (20.038-50.696)  22.233 (16.860-27.606)  16.833 (12.096-23.786) | **<0.001** |
| **AJCC 8^th^ edition**  IA  IB  IIA  IIB  III | 55.300 (38.120-72.480)  34.433 (11.144-57.722)  52.833 (NC)  49.733 (36.825-62.642)  26.667 (22.764-30.570) | **<0.001** | NC  27.633 (23.066-32.201)  NC  31.000 (23.260-38.740)  16.133 (12.982-19.284) | **<0.001** |

**Supplementary table 4.** Analysis of demographic, clinical, surgical and pathological factors associated with survival (A) and recurrence (B) in node-positive patients.

| **Variable** | 1. **Survival Analysis** | | 1. **Recurrence Analysis** | |
| --- | --- | --- | --- | --- |
|  | **Median OS, months (95% CI)** | **p-value** | **Median DFS, months (95% CI)** | **p-value** |
| **Sex**  Male  Female | 30.367 (24.095-36.638)  36.100 (27.463-44.737) | 0.905 | 20.000 (16.450-23.550)  18.233 (14.041-22.426) | 0.597 |
| **Preoperative pain**  No  Yes | 36.000 (15.283-56.717)  32.700 (26.942-38.458) | 0.581 | 19.833 (17.333-22.334)  18.967 (10.209-27.725) | 0.275 |
| **Unintentional weight loss**  No  Yes | 35.367 (27.739-42.995)  29.567 (22.268-36.865) | 0.302 | 18.400 (16.406-20.394)  21.967 (16.988-26.945) | 0.291 |
| **Preoperative jaundice**  No  Yes | 29.867 (17.127-42.606)  32.900 (27.384-38.416) | 0.921 | 31.533 (24.818-38.249)  18.400 (16.113-20.687) | **0.011** |
| **Preoperative vascular involvement**  No  Yes | 36.367 (27.504-45.230)  25.667 (19.370-31.963) | **0.006** | 21.467 (16.954-25.980)  16.867 (12.423-21.311) | **0.025** |
| **Diabetes mellitus**  No  Yes | 32.733 (26.692-38.775)  33.400 (18.992-47.808) | 0.659 | 19.367 (16.770-21.964)  20.267 (11.829-28.704) | 0.702 |
| **ASA score**  1  2  3 | 68.867 (45.027-82.706)  30.367 (24.527-36.206)  33.400 (22.869-43.931) | **0.030** | 24.000 (13.274-34.726)  19.000 (16.112-21.888)  19.900 (13.599-26.201) | 0.753 |
| **R-status**  R0  R1 | 42.467 (32.244-52.689)  27.467 (22.468-32.466) | **0.001** | 23.767 (17.274-30.259)  18.567 (16.311-20.822) | **0.023** |
| **Tumor grade**  G1  G2  G3 | NC  44.033 (33.389-54.677)  19.000 (16.043-21.957) | **<0.001** | 62.633 (10.726-114.540)  22.233 (17.256-27.211)  13.100 (9.503-16.697) | **0.001** |
| **Perineural invasion**  No  Yes | 32.733 (27.424-38.042)  27.467 (NC) | 0.447 | NC  19.733 (17.249-22.217) | 0.476 |
| **Lymph-vascular invasion**  No  Yes | 32.733 (27.423-38.044)  19.400 (NC) | 0.679 | NC  19.833 (17.344-22.323) | 0.656 |
| **Peripancreatic fat invasion**  No  Yes | 42.467 (12.544-72.389)  32.700 (27.205-38.195) | 0.983 | 33.133 (27.145-39.121)  19.733 (17.245-22.221) | 0.705 |
| **Postoperative complications**  No  Yes | 36.000 (28.443-43.557)  30.367 (23.394-37.339) | 0.826 | 20.200 (14.396-26.004)  19.367 (16.865-21.869) | 0.938 |
| **Adjuvant treatment**  No  Yes | 18.667 (4.561-32.772)  40.300 (32.529-48.071) | **0.001** | 17.500 (9.715-25.285)  20.100 (17.029-23.171) | 0.037 |
| **T-status**  T1  T2  T3 | 42.400 (29.127-55.673)  32.700 (25.441-39.959)  19.000 (12.710-25.290) | **0.001** | 30.767 (22.376-39.157)  17.933 (15.450-20.416)  11.533 (0.202-22.864) | **0.004** |
